# Disaster risk reduction communication during the Mount Semeru eruption in East Java, Indonesia

**DOI:** 10.4102/jamba.v17i1.1849

**Published:** 2025-06-11

**Authors:** Rachmah Ida, Endra Gunawan, Sri Widiyantoro, Cecep Pratama, Nuraini Rahma Hanifa, Muhammad Saud

**Affiliations:** 1 Department of Media and Communication, Universitas Airlangga, Surabaya, Indonesia; 2 Faculty of Mining and Petroleum Engineering, Bandung Institute of Technology, Bandung, Indonesia; 3 Faculty of Technical, Gadjah Mada University, Yogyakarta, Indonesia; 4 Indonesian Research and Innovation Bureau, Jakarta, Indonesia; 5 Department of Sociology, Faculty of Social and Political Sciences, Universitas Airlangga, Surabaya, Indonesia

**Keywords:** Semeru eruption, risk communication, disaster, community engagement, Mount Semeru, East Java, Indonesia

## Abstract

This research is focused on disaster risk communication management and local community engagement during the Mount Semeru eruption in 2021. The problem faced by the East Java regional government, regency governments and the regional disaster management and mitigation agency (Badan Nasional Penanggulangan Bencana [BNPBD]) is the unavailability of communication protocols and strategies in the event of a disaster and mitigation coordination for follow-up programmes. In communicating disaster risk, the government is considered most appropriate as a risk communicator.

**Contribution:**

The study examines the risk communication process carried out by the government and the risk messages it conveys and explores the perceptions of stakeholders. Furthermore, it highlights the importance of risk communication for disaster mitigation and as an early warning system and focuses on the role of community involvement in disaster mitigation efforts. The method used is descriptive qualitative with data collection techniques through a review of government documents, literature studies, direct observation by observing government programmes and in-depth interviews with 35 selected informants who live in disaster-prone areas in Lumajang and Jember regencies. The study suggests that, during the Mount Semeru eruptions, both the central and regional governments must carry out risk communication management in handling and responding to the public’s need for information related to disasters.

## Introduction

East Java is one of the volatile and crucial provinces in Indonesia that has experienced incidents of earthquakes, disasters and hydrometeorology (flooding, landslides and so forth). In 2021, a major disaster happened and caused huge impacts on several regencies such as Malang, Blitar, Lumajang, Jember and Trenggalek. These regencies experienced frequent severe incidents of disasters and volcanic eruptions. Recently, on 04 December 2021, the highest mountain and active volcano, Mount Semeru, erupted and caused damages, injuries and deaths to the surrounding villages, particularly in Lumajang regency.^1^ Before the eruption, a major disaster happened in Jember, a neighbourhood regency of Lumajang. A series of disasters that occurred in 2021 have made the East Java provincial government busy in managing the casualties and physical damages in the impacted areas.

Following some disasters, the continuing problem that has been faced by the regional governments of East Java, the regency’s authorities and a regional disaster management and mitigation body (known as Badan Nasional Penanggulangan Bencana ‘BNPBD’) is the unavailability of communication protocols and strategies in the event of disaster and mitigation coordination for follow-up programmes. The issue is that the establishment of a risk communication strategy and public communication model(s) for disaster management, mitigation and community resilience in this provincial state remains crucial. One consequence of this is the impacted communities and victims of the disaster have received late responses both from the local government and external support. Indeed, risk communication is a crucial element in disaster risk reduction and during the disaster, especially in designing or planning the protocols and implementing early warning systems for the potential victim communities. The ability to communicate hazard forecasts and risk information to directly impacted society or vulnerable communities and stakeholders is significant for effective disaster preparedness and response to reduce casualties and major impact and to minimise loss of life.

So far, the issue of risk communication and community engagement strategies in the event of geological and natural disasters in East Java, and Indonesia in general, has been understudied, particularly in the context of Indonesia. However, multiple studies have discussed the Mount Semeru eruption. For instance, factors affecting the tourist to revisit Mount Semeru during the post-2022 volcanic eruption (Susanto et al. 2024), Partial Least Squares - Structural Equation Model (PLS) model to analyse the socio-economic impacts (Kusumawardani et al. 2023), mapping a disaster-prone area (Wahyuningtyas et al. 2021), disaster mitigation and preparedness (Usman, Murakami & Kurniawan 2023), development of a disaster risk map (Bachri et al. 2024) and spatial analysis of this area (Utami et al. 2023). Therefore, the literature has not discussed the issue of risk communication in two regions or specifically on the risk management. This study is timely to investigate and assess the topic of risk communication and community engagement strategies in the event of earthquake and volcano disasters. It investigates documents and narratives of the presumed ‘risk communication’ that have been made and implemented by the provincial and local governments and disaster body (BNPB), as well as public communication model(s) and the community engagement strategies of earthquake disaster and volcano eruption in East Java. This study selected Lumajang and Jember regencies as the locus of the study following the recent disasters that occurred in these two places.

Based on these considerations, this study raises the following questions: How do the stakeholders in regional East Java perceive the importance of risk communication for disaster mitigation and as an early warning system? Are there any documents or models of risk communication that have been produced and implemented so far? What have the regional government and stakeholders in the areas of disaster learned from the recent earthquake and eruption disasters in terms of public communication, risk communication and information management? How have communities been involved and informed during the disaster event and mitigation? How can communities and the regional government communicate effectively in future disasters to reduce the impacts?

This study intends to identify the perceptions of the regional stakeholders and the communities impacted about the importance of risk communication and community engagement for disaster mitigation and as an early warning system. It is also to gain information from the local communities impacted about their engagement and if there are any strategies of the regional government to initiate community engagement for disaster mitigation. The selection of Lumajang and Jember regencies is based on the consideration that geographically, Lumajang Regency became the area that was directly affected by the eruption of Mount Semeru in 2021 and also in 2022. There is also a regional state organisation that manages disaster management that has been established by the Governor of East Java.

## Literature review

The concept of risk communication has been discussed by multiple scholars in academic literature. Until now, the definitions of risk communication are related to and utilised for the issues of public health (DiClemente & Jackson 2016), the critical role of coping strategies (Olonilua & Aliu 2025), community reliance mechanisms (Joseph et al. 2020), advancing risk communication (Stewart 2024), volcano disaster risk management (Andreastuti et al. 2023) and tsunami risk communication and management (Rafliana et al. 2022). However, specifically, only Fukushima and Tohuku have used the concept of risk communication (Walravens, O’Shea & Ahrenkiel 2022). From this literature, it can be summarised that risk communication is an exchange of information, a systematic dissemination of information and a communication process.

The term risk communication emerged for the first time in the 1970s. According to Krewski, Turner and Tyshenko (2011), risk communication as a distinct concept was formerly used in scientific literature in 1984. It was used initially in psychological research, in which the term is applied in relation to the theory of risk perception. Further, it was used to explain how persons and groups formed and maintained various views about risk acceptability. It includes several issues such as risk probability, technical information and technical risk assessments with public participation. However, in the 1990s, the term focused on public trusts, open discourse and shared decision-making processes.

Risk communication management and strategies in some research involve and relate to the social context and local community (Heath & O’Hair 2020; Kamrin 2014). Public trust, perception and reception are important aspects in the implementation of risk communication (Heydari et al. 2021). The sociocultural factors affect the transmission of risk information. The factors such as the ethnic and socioeconomic conditions of communities become significant elements in risk communication. According to Ulrich (2009), risk refers to the probability that an undesirable event, situation or condition occurs. The governments and some organisations, as well as individuals, with the conceptualisation, mitigation and management of risks, have become the principles of organising in some countries of the world. This does not mean that the red flags or uncertainty of disasters are increasing, but the government is considered to be an actor who can increase the control of institutions and society at large through good risk governance (Brown 2020). Thus, good risk governance depends on efforts to mitigate the disaster through good risk communication as well in the region.

The present study uses the definition of risk communication as stated by the World Health Organization (WHO), which is usually applied in public health emergencies. Risk communication includes the range of communication capacities required through the preparedness, response and recovery phases of a serious public health event to encourage informed decision-making, positive behaviour change and the maintenance of trust. This definition is close to the current research interest and intention. In this regard, the concepts of risk communication and community engagement are considered as the two important factors in the issue of disaster mitigation and early warning systems of disaster risk. It is viewed as the dissemination of information to the public about disaster risks and events, such as earthquakes and eruptions, and instructions on how to change behaviour to mitigate the disaster risk. It is thus a relevant issue in Indonesia since the new communication and media technologies and practices that have been advanced in the 21st century.

In addition, The World Bank (2013) reports added that the mitigation stage that needs to be carried out related to communication risk is the trust of information sources such as the government. Thus, credible sources of information will create public trust to manage the disaster mitigation strategies. In this sense, communicating a risk is a very crucial aspect that needs to be designed, regulated and implemented by the government with relevant agencies and community involvement. Boholm (2019) states that risk communication practices are very important to implement for policies and regulations that cover policy areas such as the environment, natural resource management, energy and contingency readiness. Government agencies are responsible for communicating their assessment of potential hazards and management to affected groups, stakeholders and the general public.

Until now, risk communication assessment has become a popular research issue and has been used widely in the studies of the COVID-19 pandemic outbreaks (Forster 2020; Herman 2021; Rahmanti et al. 2021). Rahmanti et al. (2021), for instance, discuss the topic of risk communication about social media use during the COVID-19 outbreak in Indonesia. The study focuses on how risk communication using social media platforms has been utilised to measure public attention on new regulations during the pandemic. However, in the Indonesian context, the topic of risk communication as an area of research is still quite rare, particularly related to disaster risk studies. Mulyasari and Shaw (2014) examined the local community response to the natural disaster and assessed the community-based society organisation’s risk communication process for disaster risk reduction in Bandung, West Java. Yudarwati, Putranto and Delmo and colleagues’ (2021) study also investigates the use of social media for disaster risk communication by the Indonesian government. It analysed the strategies of messaging on the government’s social media for disaster risk communication. The method of content analysis was applied to examine some cases. The current research focuses on the regional risk communication practices and community engagement strategies attempted to enhance disaster mitigation and early warning systems in the event of earthquake and eruption disasters.

Government communication includes communication carried out by the highest leadership or executive in the central or regional government. Executive communication is contrasted with legislative communication used to decide public policy through legal determination, and with the judiciary, whose function is to make judgements concerning disputes about the application of the law (Canel & Sanders 2013). Catastrophic events, global temperature warming and climate change, all of which stem from increased greenhouse gas (GHG) emissions, are public concerns not only in Indonesia but around the world. In response to growing public concerns about environmental risks, governments are increasingly looking for better ways to communicate risk information to every citizen and public group (eds. Covello, McCallum & Pavlova 1989).

## Research methods and design

This study uses a qualitative research approach to study risk communication and community engagement for disaster mitigation in the recent eruption of Mount Semeru disaster in East Java province of Indonesia. It interviews the regional governments of two regencies, including Lumajang and Jember, the stakeholders and the local communities impacted by the disaster. This study also applies storytelling analysis to gain information and stories from the community members regarding their experiences in participating and being involved in the process of disaster mitigation and to assess the availability and the existence of the pretended ‘risk communication’ management model and strategies and early warning system attempted both by the East Java provincial governments. This study also investigated relevant documents, public communication materials and media archives related to the disaster mitigation during the disaster.

The study also uses data from archived records. The archival records are often in computerised form, such as maps and charts of geographical characteristics of a place; lists of names and other relevant commodities; survey data; collected record data or census data; and organisational records such as charts and budgets of a given period organisation. These archival recordings are used in conjunction with other sources of information in the conduct of case studies. The archival records data collection technique was carried out by researchers because basically archival records are data sources that have a role as a valuable source of information for understanding the event. The archival records utilised in this study are Law Number 24 of 2007, Regulation of the Head of the National Disaster Management Agency Number 3 of 2008, Disaster Management from Bureau of Meteorology, Disaster, and Earthquake (BMKG), Budget Implementation Document of the Lumajang and Jember Regency Regional Apparatus Work Unit, Projection of Disaster-Prone Map of Lumajang and Jember Regencies and the handout on disaster management policies and management by Badan Penanggulangan Bencana Daerah (BPBD) Lumajang and Jember Regencies.

This study uses a purposive method to determine informants based on pre-determined characteristics and requirements. This consideration is based on the fact that these people are considered to know the most about the main issue in this study. Moreover, informants were selected based on the quality of the informants to the problems to be studied, such as individuals who experienced firsthand the events of the Mount Semeru disaster, persons directly or indirectly involved in mitigating the Mount Semeru disaster and local officials related to the Mount Semeru disaster. Interviews and storytelling are used as a data collection technique in this research in order to know the perceptions and the feelings of the local people as informants of this study. This research invited thirty five participants including fourteen villagers, seven local leaders, five key influential members in the villages, seven community members directly impacted by the Mount Semeru eruptions and two officials from the regional disaster mitigation. All the informants were recruited purposively; as for the community members, we used a snowball technique to find the people who were experienced and had information during the eruption time. The fieldwork and data collection were gathered around 7 to 8 months after the eruption in January 2023.

### Ethical considerations

Ethical clearance to conduct this study was obtained from the Universitas Airlangga Faculty of Social and Political Sciences Research Ethics Committee (No. 1190/UN3.FISIP/III/PT./2023).

## Results

The areas of this study covered several villages in Lumajang Regency, especially in villages affected by the eruption of Mount Semeru on 4th December 2021 (and exactly the same date on 4th December 2022, when Mount Semeru erupted and impacted several villages of the present study). Lumajang Regency is one of the areas located in the southern part of East Java Province, consisting of 21 districts with regional boundaries, namely the north of Probolinggo Regency, east of Jember Regency, south of the Indonesian Ocean and west of Malang Regency. Lumajang Regency has considerable potential in the agricultural and mining sectors because it is located in the southern area.

According to the data provided by Bank Pembangunan Daerah (BPD) Lumajang, there are as many as 21 districts in Lumajang that have different risks of natural disasters. Some disasters include earthquakes, tsunamis, volcanic eruptions, extreme weather, droughts, floods, flash floods, landslides, extreme waves and abrasion, and forest and land fires. On 04 December 2021 (and 04 December 2022), the Mount Semeru eruption caused large areas or locations to be affected by direct volcanic materials and ash. The following examines how local stakeholders, community movements, media and affected communities provide their perceptions of risk communication and mitigation management during the event of the Mount Semeru eruption in 2021. So, the depth and extent of understanding and knowledge of disaster management for BPBD officers and parties involved in Lumajang Regency and Jember Regency is one of the key factors in the successful implementation of disaster observation and analysis activities as well as prevention, mitigation, preparedness, early warning, emergency management, disaster rehabilitation and reconstruction. The deeper and broader the knowledge of these personnel regarding risk communication, the greater the success of disaster observation and analysis activities as well as prevention, mitigation, preparedness, early warning, emergency management, rehabilitation and reconstruction of the disaster.

## Discussion

### Government’s perceptions of the practices of risk communication management in villages

The impact of the eruption of Mount Semeru in December 2021 left significant stories of experience for the local governments and the affected communities as well as the public in general. This raises awareness of the importance of communication in disaster management:

‘The Head of Prevention and Preparedness of BPBD Lumajang Regency stated that providing understanding to the community regarding disaster knowledge is not easy; it takes extra effort because usually something new is difficult for the community to accept, especially for people whose education level is low and who have never experienced a disaster. It is usually even underestimating. Those are all the challenges that have always been faced by the governments. For this reason, the regional government through BPBD in both regencies continues to provide assistance and advice for villagers.’ (Head BPBD, Male, July, 2022).

**FIGURE 1:**
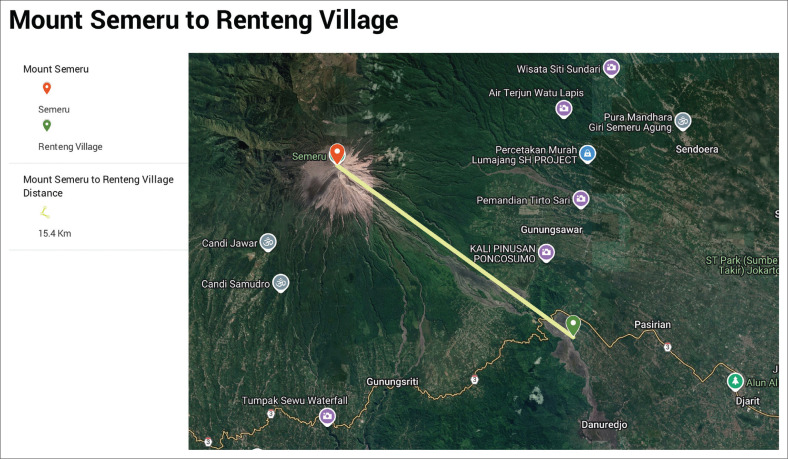
Distance from Mount Semeru to Renteng village.

**FIGURE 2:**
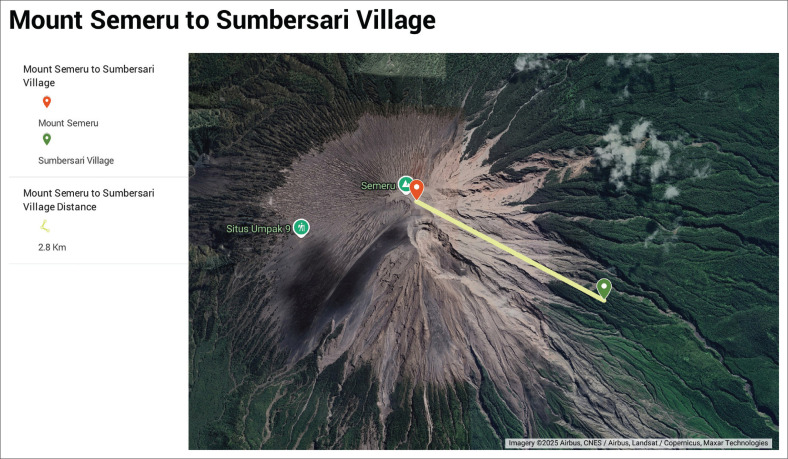
Map distance between Sumbersari village and the top of Mount Mahameru.

**FIGURE 3:**
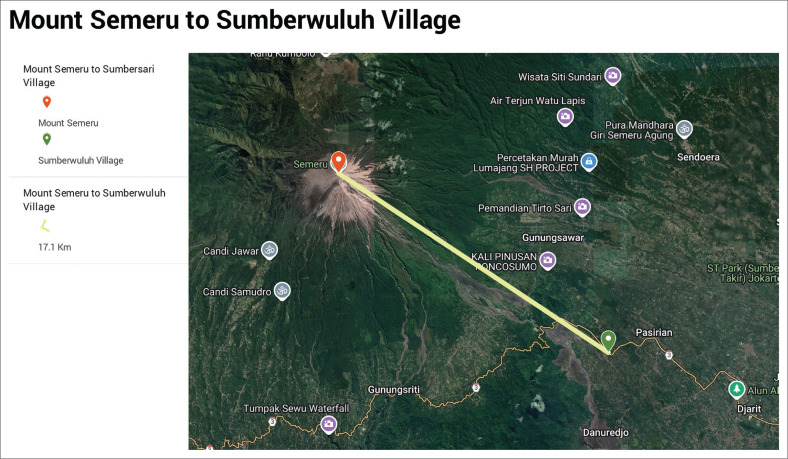
Distance from Sumberwuluh village to Mount Semeru.

Figure 1, Figure 2 and Figure 3 are the views of the distances between Mount Semeru and the villages that experienced quite devastating conditions during the eruption of Semeru in 2021.

Both regency governments, Lumajang and Jember, assert that they have done a series of socialisation and workshops for disaster management and mitigation for the villagers. These regional governments have also trained volunteers to assist and provide support for villagers in the event of eruptions. However, there still is a barrier to enhancing the ability of the villagers to mitigate in the event of disaster because of the educational gap that still persists and the cultural beliefs held by the people, such as that disaster is destined from God, so if people die, it is because of his or her destiny. Such cultural beliefs sometimes make it difficult to continue training villagers with evacuation and mitigation procedures, particularly with risk communication procedures, where villagers still believe in their family members rather than outsiders or the government officials and volunteers. According to the officer of Lumajang’s Disaster Agency in a personal interview, the organisation has trained volunteers related to disaster knowledge. The volunteers serve as early detection of potential disasters that may occur and report them directly to the agency. The agency provides socialisation to the community and has carried out disaster management procedures.

One of the key points of concern related to communication in disasters is the issue of uncertainty. According to Frank Dance (cited in Littlejohn 2006:7), one of the important aspects in communication is the concept of reducing uncertainty. Communication itself arises because of the need to reduce uncertainty in order to act effectively and protect or strengthen the ego concerned in interaction between the individuals or in a group. In disaster management, accurate information is needed by the public and private institutions that have concern for disaster victims. Conveyed by Lumajang’s BPBD Field Team, the agency is actually very active in monitoring the latest condition of Mount Semeru.

The researchers went to Curah Kobokan post and were able to talk to local communities affected by the Semeru eruption. The researchers also observed that there were about 20 refugee families who have now returned to occupy their homes in Kamar Kajang village and Curah Kobokan hamlet. Based on the disaster-prone map, both areas are declared to be no longer able to be occupied as locations where people could live. From interviews with the people of Curah Kobokan village, we gained a typical comment that people did not want to move out of their village because of preserving their memories, family heritage assets and the vulnerability of them to own a new house or shelter. The villagers were aware of the danger and risk of eruption as they live on the edge of the mountain. They see that eruption is just a small liquid of lava and do not perceive it as dangerous because the villagers, from their childhood to elders, have seen the light of lava liquid almost every day within their entire lives. The villagers have been collecting some clues if the volcano will be closer, and they could feel the air and environment surrounding them. By ‘reading’ the clues, the villagers will then move out of their place.

Risk communication is an integral strategy of disaster management and continues in the practice of risk analysis. Ideally, all stakeholders should be involved from the beginning so that they understand each stage of the risk assessment. This will help ensure that the logical conditions, significance and limitations of risk assessment are clearly known by all stakeholders, including information from stakeholders that are crucially important. In addition, there was a failure of both the government and its related agency of disaster mitigation to provide information in the event of the Semeru eruption in December 2021, especially in Lumajang. An interview with the officer of the Center for Volcanology and Geological Disaster Mitigation (hereafter, Pusat Vulkanologi dan Mitigasi Bencana Geologi [PVMBG]) showed that there were differences in communication patterns carried out by relevant authorities during the eruption of Mount Semeru in 2020 and 2021.

In the 2020s incident, as also conveyed by the community, there has been information circulating in the community regarding the early warning so that there were anticipatory steps taken. Meanwhile, in 2021’s incident, there was absolutely no information issued by the relevant authorities so that the disaster had a tremendous impact on the people of Semeru. From this statement, researchers also double-checked and searched through the Lumajang’s BPBD website, and the results showed at the time of the 2020 eruption, BPBD actively conveyed information through its website, while the eruption that occurred in 2021 did not find any early warning efforts submitted by the relevant authorities. In our interview with the officer, the female officer said, in 2020, we at PVMBG used two methods: visual graphics and Closed-Circuit Television (CCTV) technology. It happened that the previous day’s monitoring was to be visually covered with fogs so that it could not be monitored with visible sight and seismic activity as usual. At that time, hot cloud avalanches had occurred for a week, but in terms of seismic activity, it was not very high:

‘We followed last (2020) year incident, as happened again in 2021 eruption, we were there (at Semeru), but it was no early warning issued, suddenly it turned out that the incident happened quite big.’ (BPBD Officer, Female, July 2022)

Risk communication serves to ensure or give confidence to the party regarding ongoing information, that if there is new and relevant information to people’s lives, the people who obtain the information can use it in their daily lives as a standard procedure for mitigation so as not to miss information. Communication can also build cooperation and a social radar between people and facilitate people for safety and resilience procedures. The existence of communication as a social radar is expected to be an extension of the government’s authority to inform or be aware of a disaster. The failure to convey information regarding the activities of volcanoes and disasters will cause large impacts for the humans and natural disasters in the country. The ignorance and inconsistency in implementing the early warning system and procedure have paid off with the serious consequences in the eruption of Mount Semeru in 2021.

### The views of villagers on risk communication

From the results of our fieldwork, there are almost similar understandings in the community around Mount Semeru in looking at the meaning of disaster risk communication. In terms of terminology used, villagers do not understand, but in substance, they understand what disaster risk communication is, actually. Their understandings were obtained from the community from various aspects, namely ‘information’, ‘socialisation’, ‘regulation’ and ‘order’. The elaboration of the understanding of the community around Mount Semeru regarding disaster risk communication shows that the ability of the majority of speakers is almost the same in capturing the meaning. Another aspect is the provision of information about the condition of Mount Semeru; it is also the provision of information about what actions should be taken by the community.

For the community, risk is perceived as a small consequence if the impact of the risk has been known or people are used to it (habituation) with the risk. During the interviews with villagers, we found that for people affected by volcanic eruptions, it may not be considered a big risk because the lava flood and earthquake occur all the time and they have gotten used to the so-called ‘risks’. The villagers will consider a risk to be high if they feel that they are not familiar with the risk. However, the public really needs actual information about volcanic and seismographic activities, and access to information is also needed to minimise the high risk:

‘One of the villagers told his story that when the Semeru eruption occurred in December 2021, he and his family and neighbours felt unusual sounds and particular signs related to the volcano, but they ignored it because they did not receive any information from relatives, the village head and other authority officers. ‘Our knowledge is low, we did not have high education, so we take it for granted, because usually once lava floods are commonplace. Since I was small, we got used to seeing that incident and we were fine. But last December (2021), we didn’t expect it, there was no preparation, even though actually if you say you feel it?, we feel it, because it is incandescent at night, but there is no info about anything.’ (Villager, Male, July 2022).

The local residents recognise that the eruption of Mount Semeru is common every year, and they are used to and very familiar with the condition. Local wisdom around Mount Semeru has actually been preserved for a long time; the local community is very familiar with the characteristics; even though there are many eruptive events that occur every year, they all do not bring casualties. There are several guidelines or technical instructions for identifying when an eruption will occur, such as the following: The odour of sulphur gas spread into a distance of more than one kilometre, which effects the colour of the crater smoke. It changes from white to greyish, a noise in the stationary fire point, avalanche of rocks (incandescent lava), a light ash rain and the crater lake water turns a cloudy colour. If these signs are visible to the locals, they then prepare for evacuation, but only if the ash clouds are bigger and the lava floods reach their place.

Instructions or guidelines as mentioned above are not visible at the time of the eruption that occurred on Saturday, 04 December 2021, at 15:20 Western Indonesian Time (WIB). According to the recognition of the local people, the eruption incident at that time was very different from the previous incident in 2020, and it was alarming. In discussion with a volcanologist from BPBD who shared that, the volcanic eruptions can be caused by three factors: firstly, because of the buoyancy of the magma; secondly, pressure from dissolved gases in the magma, and lastly pressure on the chamber lid. Further, other factors including heavy rainfall and accumulation of previous eruptions eroded by water have become a cause of volcano eruption in Semeru. All these scientific explanations seem to be not understood by and not compliant with the conventional knowledge held by the local residents of Semeru.

From the details mentioned above, we know that risk communication during pre-disaster times is very important to be carried out. Informants shared that, natural signs or symptoms before a disaster can helpful to sense the disasters, but it is also important to confirm and reinforce those observations with information from authorities such as BPBD and PVMBG. The government sometimes seems slow and lacking in the process of coordinating and distributing information. In fact, when viewed in this phenomenon, many of the people have been able to be active in helping the affected communities. This proves that the role of social radar in disaster coverage is good enough to bring a significant impact on all stakeholders. Risk communication can serve as the basis of communication actions or activities being a tool for regulating or controlling members of society or communities.

### Regional risk communication management and community involvement

Disaster communication and information systems are important elements in the disaster cycle to prepare protection in the pre-disaster, disaster and post-disaster stages, especially in disaster-prone areas, such as Mount Semeru in Lumajang Regency. The system overcomes the current bottleneck for an integrated information system. If communication is hampered, the survivor’s information and data become invalid and unclear. As a result, the distribution of assistance that is not on target delays medical actions to victims, thereby reducing the risk of communication. During disasters and post-disasters, local governments are trying to build a more intense risk message so that the community can always work hand in hand in helping others and in the spirit of handling volcanic disasters. Local governments visualise the message via infographics on various communication channels owned by local governments, both through the BPBD website and social media of the Lumajang regency government. Based on the findings of researchers in the field, the implementation of disaster communication management in the 2021 Mount Semeru disaster was not preceded by planning. This is because the eruption of Mount Semeru was not predicted before. The sudden disaster prompted the Lumajang Regency Government to immediately take quick action when the event occurred without any prior planning. The quick action taken by the government based on the direction of the Lumajang Regency at that time was to evacuate residents around the slopes of Mount Semeru by involving the agencies in the Satuan Kerja Perangkat Daerah (SKPD) (Regional Apparatus Work Unit) of Lumajang Regency, the Indonesian military and the police. Ironically, during the eruption in 2021, according to one of the village heads interviewed in this study, there was not any single system operational procedure available that could be used as guidance to evacuate the people. He only used the Lumajang government’s instruction and centralised coordination. An excerpt from an interview with the village head shows that disaster communication management carried out without prior planning caused the evacuation process, with many residents being scattered because the evacuation sites were not determined based on village origin or based on certain categories. This caused a new problem that made the village head check the refugee camps to find out the whereabouts of his villagers.

The evacuation of residents carried out by the Lumajang Regency government as a quick action shows that the mode of communication messages is a one- way or top-down method and indicates communication by the management to the local government. But, this information was not timeously shared to the villagers. This miscommunication occurs because of messages being delivered late to the communication target (villagers). In the end, it caused the evacuation process to not take place optimally.

**FIGURE 4:**
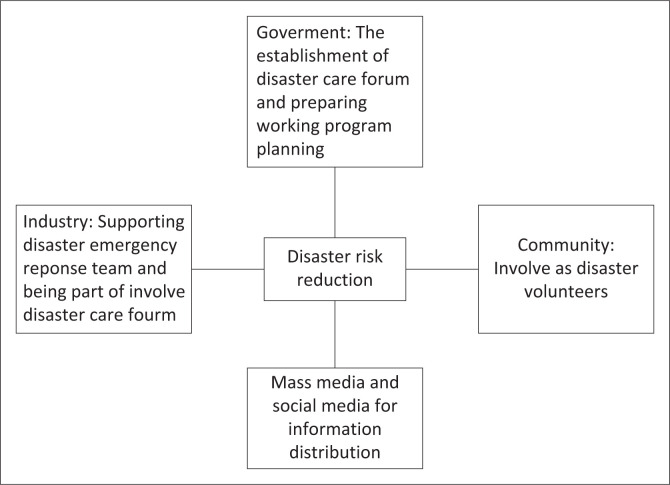
Risk management model.

Nevertheless, in terms of community engagement, both the regional governments of Lumajang and Jember have initiated a stakeholders forum called ‘Forum Peduli Bencana’ (locally known as Disaster Care Forum or FPB). FPB is a professional non-profit institution, a forum for meeting, assessing, researching and developing expertise in community-based integrated disaster management, both individually and institutionally. These forum members come from various backgrounds, including practitioners, scientists, academics, professionals, humanitarian institutions, Non-Governmental Organisation (NGOs), bureaucrats, volunteers and donors as well as contributors who have an interest in events, handling and reducing disaster risk with a touch of local wisdom and are oriented towards sustainable development and always put disaster victims as subjects and, at the same time, their main targets. Apart from this forum, people living around Mount Semeru have also established a volunteer group called ‘Laskar Semeru’ (Semeru’s Army), which contains young people from the Sumbersari and Supiturang villages. This army has created live monitoring technology for Semeru’s activities post-eruption in 2021. These CCTVs have been utilised as early warning systems to monitor cold lava floods, lava rain flows and any sign of eruption from Semeru, independently as a community’s initiative, because they realised that it was impossible to ask for the government’s funding allocation to cover the costs of the CCTVs and the operation activities. There are various actors and stakeholders involved in the risk management disaster mitigation during the Semeru eruption in 2021, just as shown in Figure 4.

However, during our study in two regencies, we did not find any official risk management model prepared. We also found that the difficulty in providing the risk management model is the lack of technological knowledge of the communities, and not all local residents or villagers get accustomed to the disaster information technology system. Many of the villagers continue to rely on the information from key persons in the village, such as the village administrative head, religious leaders and some elders who are believed by the common residents:

‘The residents here mostly work as farmers, farming labors, and are attained only education in elementary school. If there is information about disasters, they follow my advice, but I cannot control the entire villagers because they [*are*] many and difficult to be reached individually. So far, there has never been a home visit regarding counseling activities about the eruption disaster of Mount Semeru attempted by the regional government and its agencies. I, personally, often participate in field rehearsals together with elite figures and regional military.’ (Village Head, Male, July 2022)

Indeed, an ideal condition where people already have the knowledge, understanding, skills and concerns of things related to disasters, this is a concept of disaster aware communities, that way they have the awareness to behave and adapt in disaster-prone areas as well as possible. The concept of disaster aware communities is essential for the community understanding about the skills and concerns of disasters. Risk communication is an important process for gathering opinions and disseminating information about risks to stakeholders. This process is an integral aspect of the risk management process, which requires various forms of communication and information activities at different stages and levels directed towards the target group of specific risk stakeholders (Ndlela 2019). Risk communication is a strategic management activity that involves the ability to effectively communicate the nature and magnitude of risk to internal and external stakeholders. It is important for risk management to not only recognise the problem but also communicate the risk to key stakeholders. There are four areas that distinguish risk communication: firstly, information and education, where people are informed and educated about risk; secondly, it stimulates behaviour change and takes protective measures through risk reduction by influencing audience perception; thirdly, disaster warning and emergency information, which provides instructions and guidance during disasters and emergencies; and lastly, information exchange and general approaches to risk issues, involving the public in the risk management process. Different types of risks will require different forms of risk communication (Ndlela 2019).

### Lesson learned: community involvement in risk communication and early warning

Again, the purpose of risk communication may also differ from technical communication. In dangerous situations, such as floods and tornadoes or other catastrophic events stemming from climate change, government risk communication should motivate audiences to act (Lundgren & McMakin 2013). In other situations, the goal is more appropriate to inform or encourage agreement on a risk solution. Risk communication more often involves two-way communication; that is, the organisation manages the risk and the audience conducts a dialogue (Lundgren & McMakin 2013). In technical communication, most efforts are designed to disseminate information but not to receive information back from the audience or to include the audience in the decision-making process (Lundgren & McMakin 2013).

One of the objectives of risk communication is to provide meaningful, relevant and accurate information in clear and easy-to-understand terms to a specific audience in order to increase awareness and understanding of various specific issues that must be considered by all participants during the risk analysis process. In addition, it also improves consistency and openness in risk management decision-making and its implementation; provides a safe foundation for understanding proposed or implemented risk management decisions; and improves the overall effectiveness and efficiency of the risk analysis process. Finally, it contributes to the development and delivery of effective information and education programmes if both of these are selected as risk management options.

From our fieldwork in Mount Semeru areas, we believe that risk communication in regional Indonesia is still preoccupied, unrecognised and closely related to the exercise of power of the regional authorities. This finding is quite different from the idealised notion of disaster risk communication, as Brown, Moerman and Broer in Patrianti et al. (2020) stated that risk is used to describe what is considered a current phenomenon or something new. So far, governments and several world organisations, through their risk management, have become examples of several world countries that can control and carry out good risk governance. In dealing with emergencies during eruptions, the government is an important source of information in communicating risk messages. Risk communication is considered a prerequisite for disaster preparedness, response and recovery. It involves exchanging information among stakeholders about disaster conditions and related risks to mitigate their impact. Even after a disaster, risk communication is needed for a message of sustainability (Karis & Cochran 2019).

## Conclusion

The current research found particular findings that distinguish the practice of risk communication management and community engagement in the regional Indonesia, that is, East Java province, which continues to be limited and rare in being studied. This study thus confirms, although there is not any established risk communication strategy model developed and used by the regional government and its apparatuses, the so-called ‘risk communication’ practice has been utilised and is perceived as very important by stakeholders, especially the East Java provincial government, two regional governments (Lumajang and Jember), the Regional Disaster Management Agency and any related organisations to disaster mitigation and the local community, to provide information to the public regarding the necessity of preparedness and the kind of preparations that should be made when a disaster occurs. We gathered stories and information from the informants for this research and understand that the notion of risk communication includes the following: risk communication acts as a social radar; risk communication as a means of socialisation; risk communication as an effort to increase the adaptive capacity of survivors; and, lastly, risk communication serves as management in handling disaster in the community.

## Limitations

There are few limitations of this study. The findings of the study are based on Mount Semeru, but this does not fully represent the risk communication in other areas. Further, the data presented in the study were collected from the informants, and hence data from the officials from the government side could strengthen the narratives. Lastly, the study does not examine the role of technology or social media tools in risk communication.

## Practical recommendations

There are three main recommendations from this study. Firstly, it suggests designing risk communication protocols, that is, visual-based alert system (colour, warnings by flag), strengthening a better radio system and regular pre- and post-messages for the updates. Secondly, challenges to adopt the learning models, storytelling and role-playing, engagement of local influencers, that is, religious teachers and Rukan Warga (RW) and Rukan Tetangga (RT), and infographic information would be useful. And, lastly, it suggests engagement of local or volunteer-based disaster communities to facilitate two-way communications, validate or document traditional early warnings or sign and co-production of knowledge in disaster planning.

## Footnotes

1 When this paper was written, Mount Semeru had just erupted again on the 4th of December 2022, with spewing a cloud of ash for 15 km into the sky and forcing the evacuation of nearly thousands of people.
